# Risk factors for persistent high-risk HPV infection and recurrence burden of vaginal infections in women with cervical low-grade squamous intraepithelial lesions: a retrospective cohort study

**DOI:** 10.3389/fcimb.2026.1983448

**Published:** 2026-10-22

**Authors:** Ru Bai, Zishan Tang, Cunwei Ji, Lingling Tang, Yan Liu, Mingyong Luo

**Affiliations:** 1 Department of Clinical Laboratory, Guangdong Women and Children Hospital, Guangzhou, China; 2 Graduate School, Guangzhou Medical University, Guangzhou, China

**Keywords:** aerobic vaginitis (AV), bacterial vaginosis (BV), high-risk human papillomavirus (HPV), persistent infection, vaginal microbiota (VMB)

## Abstract

**Background:**

Persistent high-risk human papillomavirus (HR-HPV) infection drives cervical carcinogenesis, yet factors determining persistence in women with low-grade squamous intraepithelial lesions (LSIL) remain incompletely characterized. The longitudinal recurrence burden of vaginal infections including aerobic vaginitis, bacterial vaginosis, and vulvovaginal candidiasis in this population has not been systematically quantified.

**Objective:**

To identify independent risk factors for persistent HR-HPV infection and evaluate the recurrence burden of aerobic vaginitis, bacterial vaginosis, and vulvovaginal candidiasis in women with cervical LSIL.

**Study design:**

This retrospective cohort study included 3,563 women with cervical LSIL who underwent concurrent vaginal microecology testing, HPV genotyping, and cervical cytology at Guangdong Women and Children Hospital (January 2021–August 2025). Patients were classified as persistent (n=919; ≥365 days with the same HR-HPV subtype) or transient (n=2,644; >30 days and <365 days). Multivariate logistic regression identified factors associated with persistence. Negative binomial regression assessed recurrence burden, expressed as incidence density per 100 person-years.

**Results:**

After adjustment, age (per 5 years: OR = 1.13, 95% CI: 1.09–1.17, P<0.001), HPV-52/58 (OR = 1.65, 95% CI: 1.38–1.98, P<0.001), and multiple HR-HPV infections (OR = 1.50, 95% CI: 1.24–1.81, P<0.001) independently predicted persistence. Bacterial vaginosis (OR = 0.69, 95% CI: 0.55–0.88, P = 0.002) and intermediate bacterial vaginosis (OR = 0.66, 95% CI: 0.52–0.84, P<0.001), and vulvovaginal candidiasis (OR = 0.75, 95% CI: 0.58–0.96, P = 0.024) showed inverse associations. Among persistently infected women, aerobic vaginitis had the highest recurrence burden (35.77 per 100 person-years, 95% CI: 33.17–38.53), exceeding bacterial vaginosis and vulvovaginal candidiasis. Persistent infection was associated with TCT abnormalities ≥ASC-US (OR = 1.63, 95% CI: 1.40–1.91, P<0.001) and LSIL+ lesions (OR = 1.48, 95% CI: 1.25–1.75, P<0.001).

**Conclusion:**

HPV-52/58, multiple HR-HPV infections, and advancing age significantly increase persistence risk in LSIL patients. The inverse associations of bacterial vaginosis and vulvovaginal candidiasis with persistence are likely non-causal and should be interpreted with caution. The high aerobic vaginitis recurrence burden underscores the need for integrated management addressing both viral persistence and vaginal dysbiosis. These findings support genotype- and microbiota-informed risk stratification.

## Introduction

Cervical cancer remains a leading cause of cancer-related morbidity and mortality among women worldwide. China alone reported approximately 151,000 new cases and 56,000 deaths in 2022 (Han et al., 2024). Persistent infection with high-risk human papillomavirus (HR-HPV) is the primary driver of cervical carcinogenesis, accounting for over 99% of cases (Sung et al., 2021; IARC, 2012; Liao et al., 2020). Although 70–90% of HR-HPV infections resolve spontaneously within 1–2 years, 10–20% persist beyond 12 months, marking a critical transition from low-grade squamous intraepithelial lesions (LSIL) to high-grade lesions and invasive cancer (World Health Organization, 2021; Bi et al., 2022). Standardized management pathways for LSIL patients remain lacking, and determinants of persistence versus clearance are incompletely understood (Zhao et al., 2022). Among Chinese women, HPV types 52 and 58 are particularly prevalent and associated with lower clearance rates, yet their role in persistence risk requires further quantification (Li et al., 2025a; Liu et al., 2025).

Emerging evidence implicates vaginal microecology as a key modifier of HPV infection dynamics (Cui et al., 2025; Kazlauskaitė et al., 2025; Dong et al., 2026; Žukienė et al., 2025). Lactobacillus-dominated vaginal communities confer protection through lactic acid production, hydrogen peroxide secretion, and immune modulation. In contrast, dysbiotic states, particularly bacterial vaginosis (BV) and aerobic vaginitis (AV), are associated with increased HPV susceptibility and delayed viral clearance (Dong et al., 2022; Amabebe et al., 2025; Hou and Guo, 2024). Recent meta-analyses confirm that bacterial vaginosis-associated anaerobes such as Gardnerella and Prevotella increase in abundance with advancing cervical lesion severity (Peng et al., 2025; Zhang et al., 2025a; Zhang et al., 2025b; Yang et al., 2025; Ye et al., 2025). However, most existing studies are cross-sectional and cannot establish temporal relationships between dysbiosis and persistence. Furthermore, the longitudinal recurrence burden of different vaginal infection types (aerobic vaginitis, bacterial vaginosis, and vulvovaginal candidiasis) in persistently HPV-infected women has not been systematically quantified, representing a significant knowledge gap for clinical management (Liang et al., 2025).

In this retrospective cohort study of 3,563 women with cervical LSIL, we aimed to: (1) identify independent risk factors for persistent HR-HPV infection, focusing on HPV genotype, age, and vaginal microbiota parameters; and (2) quantify the longitudinal recurrence burden of aerobic vaginitis, bacterial vaginosis, and vulvovaginal candidiasis in persistently infected patients. By integrating clinical data with rigorous statistical methods, we sought to generate evidence to inform personalized surveillance strategies and improve risk stratification for LSIL patients.

## Materials and methods

### Study design and population

This retrospective cohort study was conducted at Guangdong Women and Children Hospital, a tertiary referral center in Guangzhou, China. We analyzed data from women who visited between January 1, 2021 and August 31, 2025 and concurrently underwent vaginal microecology testing, HPV genotyping, and cervical cytology (TCT). The study protocol was approved by the Institutional Ethics Committee of Guangdong Women and Children Hospital. All data were de-identified prior to analysis, and the requirement for written informed consent was waived.

Inclusion criteria were: (1) women aged ≥16 years (no upper age limit was applied; the observed age range was 16–86 years for the full cohort and 18–86 years for the persistent group; see **Table 1**); (2) history of sexual activity; (3) not in menstrual period or with active vaginal bleeding; (4) complete medical records without missing key variables; and (5) completion of all three tests.
Exclusion criteria included: (1) pregnancy, lactation, or menstruation; (2) use of antibiotics or vaginal preparations within 2 weeks before testing; (3) acute reproductive tract infections; (4) history of uterine surgery, hysterectomy, or pelvic radiotherapy/chemotherapy; (5) malignant tumors; and (6) underlying diseases including diabetes, cardiovascular disease, hematological disorders, or immune system diseases.

**Table 1:** Baseline characteristics of the study population.

Variable	Level	Persistent (n=919)	Transient (n=2,644)	*P*
Age (years, mean ± SD)		37.74 ± 10.48	35.48 ± 9.83	<0.001
HR-HPV subtype	HR-HPV-16/18	195 (21.2%)	585 (22.1%)	<0.001
	HR-HPV-52/58	472 (51.4%)	1,063 (40.2%)	
	Other HR-HPV	252 (27.4%)	996 (37.7%)	
HR-HPV infection number	Single	680 (74.0%)	2,140 (80.9%)	<0.001
	Multiple	239 (26.0%)	504 (19.1%)	
Mixed infection	No	749 (81.5%)	2,193 (82.9%)	0.346
	Yes	170 (18.5%)	451 (17.1%)	
AV	No	703 (76.5%)	1,987 (75.2%)	0.44
	Yes	216 (23.5%)	657 (24.8%)	
BV	Normal	632 (68.8%)	1,683 (63.7%)	0.018
	Intermediate	139 (15.1%)	452 (17.1%)	
	BV	148 (16.1%)	509 (19.3%)	
VVC	No	831 (90.4%)	2,286 (86.5%)	0.002
	Yes	88 (9.6%)	358 (13.5%)	

Data are presented as mean ± standard deviation for continuous variables and n (%) for categorical variables. P-values were calculated using t-test for continuous variables and chi-square test or Fisher's exact test for categorical variables. HR-HPV-16/18 includes HPV types 16 and 18; HR-HPV-52/58 includes HPV types 52 and 58; other HR-HPV includes types 31, 33, 35, 39, 45, 51, 56, 59, and 68. Multiple HR-HPV infection was defined as detection of two or more high-risk types concurrently. Mixed infection was defined as concurrent HR-HPV and low-risk or intermediate-risk HPV infection. bacterial vaginosis classification: normal (Nugent score 0–3), intermediate (Nugent score 4–6), bacterial vaginosis (Nugent score 7–10). HR-HPV, high-risk human papillomavirus; AV, aerobic vaginitis; BV, bacterial vaginosis; VVC, vulvovaginal candidiasis. Age range: 16–86 years for the full cohort; 18–86 years for the persistent group.

### Group definitions

Patient trajectories were reconstructed using hospitalization numbers, visit dates, and HPV test results. The index date (t0) was defined as the first HR-HPV-positive test. Participants were classified into:

Persistent HR-HPV infection group (n=919): Women with ≥2 positive tests for the same HR-HPV subtype, with interval between first and last positive test ≥365 days.

Transient HR-HPV infection group (n=2,644): women positive for the same HR-HPV subtype at baseline and negative at the next test, with an observed infection duration >30 days and <365 days. The 30-day minimum interval was applied to avoid classifying two tests performed within the same clinical episode as separate events.

The 365-day threshold is consistent with the widely used ≥12-month definition of HR-HPV persistence (IARC, 2012; World Health Organization, 2021; Koshiol et al., 2008; Rositch et al., 2013). Testing was visit-driven rather than protocol-driven; testing frequency and intervals are reported in **Supplementary Table S7** and the study timeline is shown in **Supplementary Figure S3**.

### Laboratory methods

HR-HPV genotyping was performed using real-time fluorescent PCR (Kairui Biotechnology, China), which detected 15 high-risk types (16, 18, 31, 33, 35, 39, 45, 51, 52, 56, 58, 59, 68), low-risk types (6, 11, 42, 43, 44), and intermediate-risk types (53, CP8304, 73, 82). Multiple HR-HPV infection was defined as concurrent detection of two or more high-risk types.

Vaginal microecology was evaluated using the SHTARS test kit with the Comet microscopy system (Shidasi Biotechnology, China), in accordance with the Chinese Society of Laboratory Medicine guidelines for standardization of vaginal secretion clinical testing (Chinese Society of Laboratory Medicine, Hematology and Body Fluid Group, and Chinese Society of Obstetrics and Gynecology, 2023). This automated platform integrates morphological assessment with functional biomarker detection, with bacterial vaginosis classification algorithmically derived from the Nugent scoring criteria and aerobic vaginitis diagnosis based on the Donders criteria, consistent with the expert consensus on clinical application of vaginal microecology evaluation (Chinese Society of Obstetrics and Gynecology, 2016). A validation study comparing this system with manual microscopy demonstrated a concordance rate of 90.8% (Kappa=0.861) for bacterial vaginosis diagnosis and 94.5% (Kappa=0.875) for fungal detection (Wang et al., 2021b). The use of this standardized system is further supported by recent expert consensus on vaginal microecology evaluation (Clinical Laboratory Science and Technology Translation Professional Committee, 2026). Nugent scores of 0-3, 4-6, and 7-10 were classified as normal, intermediate bacterial vaginosis, and bacterial vaginosis, respectively, in accordance with the Chinese guidelines for diagnosis and treatment of bacterial vaginosis (Chinese Society of Obstetrics and Gynecology, 2021). Aerobic vaginitis was diagnosed at scores of 3 or higher, and vulvovaginal candidiasis was identified by the presence of Gram-positive fungal spores, blastospores, or pseudohyphae. Vaginal pH ≥4.6 was considered elevated relative to the normal range of 3.8–4.5. Functional assays included assessments of hydrogen peroxide production, sialidase, and leukocyte esterase activities.

Cervical cytology was classified according to the Bethesda System as NILM (negative for intraepithelial lesion or malignancy), ASC-US (atypical squamous cells of undetermined significance), ASC-H (atypical squamous cells—cannot exclude HSIL), LSIL (low-grade squamous intraepithelial lesion), HSIL (high-grade squamous intraepithelial lesion), or SCC (squamous cell carcinoma), with abnormalities defined as TCT (ThinPrep cytology test) results of ASC-US or higher.

### Statistical analysis

Statistical analyses were performed using SPSS 26.0 (IBM, Armonk, NY, USA) and R version 4.3.2 (R Foundation, Vienna, Austria). Continuous variables were expressed as mean ± SD or median (IQR), and categorical variables as frequencies (percentages). Between-group comparisons used t-tests, chi-square tests, or Fisher’s exact test.

Univariate and multivariate logistic regression models were constructed with persistent HR-HPV infection as the binary outcome. Variables with P<0.10 in univariate analysis were included in the multivariate model, with results reported as odds ratios (OR) and 95% confidence intervals (CI).

In the persistently infected population, recurrence burden was assessed using incidence density (episodes per 100 person-years), with person-time calculated from the first positive HR-HPV test to the last recorded visit. A new episode of aerobic vaginitis, bacterial vaginosis, or vulvovaginal candidiasis was defined as a positive test occurring >30 days after the previous episode of the same type. Because a 30-day threshold may conflate treatment failure with true recurrence, sensitivity analyses using >60-day and >90-day thresholds were performed (**Supplementary Table S9**). Consistent with the commonly used framework, a positive diagnosis within 22–30 days after treatment initiation was considered treatment failure, and a positive diagnosis thereafter was considered recurrence (Bradshaw et al., 2006; Vodstrcil et al., 2021; Workowski et al., 2021). Negative binomial regression was employed to evaluate factors associated with recurrence burden, reporting incidence rate ratios (IRR) and 95% CI.

To test robustness, we conducted restricted analyses including: (1) follow-up ≥180 days; (2) follow-up ≥365 days; (3) HR-HPV-only infections; (4) single HR-HPV infections; (5) persistent vs. single-positive; and (6) transient vs. single-positive comparisons.

All tests were two-sided, with P<0.05 considered statistically significant.

To address potential immortal time bias arising from the ≥365-day persistence definition, the index date (t0) was used as the start of person-time for both groups, and pre-specified restricted analyses were performed (follow-up ≥180 days, follow-up ≥365 days, HR-HPV-only infections, single HR-HPV infections, persistent vs. single-positive, and transient vs. single-positive comparisons; **Supplementary Table S6**; **Supplementary Figure S2**).

In addition, to evaluate the inverse bacterial vaginosis/vulvovaginal candidiasis associations, we (1) compared testing intervals between bacterial vaginosis-positive and -negative women, (2) performed a sensitivity analysis excluding patients with short-interval re-testing as a proxy for immediate post-diagnosis management, and (3) assessed the temporal ordering of bacterial vaginosis/vulvovaginal candidiasis diagnoses relative to the first HR-HPV-positive test (**Supplementary Table S8**).

## Results

From 211,667 visit records representing 120,475 individual patients, we applied exclusion criteria to derive a final analytical cohort of 3,563 women. Among these, 919 (25.8%) met criteria for persistent HR-HPV infection, while 2,644 (74.2%) had transient infection (**Figure 1**). The median duration from first to last positive test in the persistent group was 595.0 days (IQR: 459.0-839.5), with a minimum of 365.0 days. Testing was visit-driven, and the median number of tests and median testing interval were 5 (IQR 4-8) and 184 days (IQR 122-282) in the persistent group, versus 4 (IQR 3-5) and 96 days (IQR 45-167) in the transient group (both P<0.001; **Supplementary Table S7**). Median follow-up was 721 days (IQR 490-1002) and 195 days (IQR 80-386), respectively (**Supplementary Table S7**). The study design and testing timeline are shown in **Supplementary Figure S3**.

**Figure 1:**
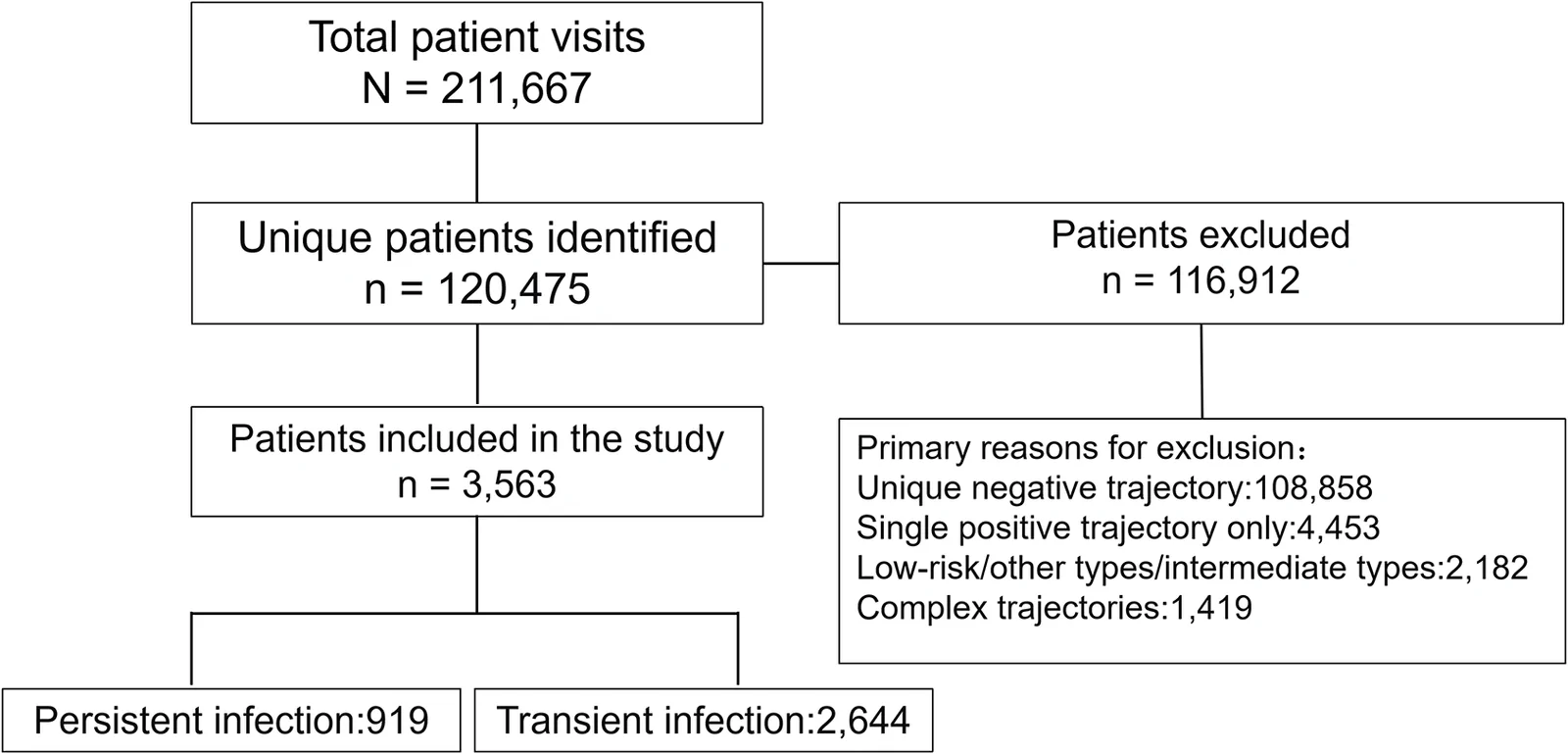
Study participant flowchart. From 211,667 visit records of 120,475 individual patients, 3,563 women met the inclusion criteria after sequential exclusions. Patients were classified into persistent HR-HPV infection group (n=919, defined as ≥2 positive tests for the same HR-HPV subtype with interval ≥365 days) and transient HR-HPV infection group (n=2,644, defined as an observed infection duration >30 days and <365 days). HR-HPV, high-risk human papillomavirus.

Baseline characteristics are summarized in **Table 1**. Patients with persistent infection were significantly older (mean 37.74 ± 10.48 vs. 35.48 ± 9.83 years, P<0.001). The distribution of HR-HPV subtypes differed significantly between groups (P<0.001). HPV-52/58 was more prevalent in the persistent group (51.4% vs. 40.2%), while other HR-HPV types were less common (27.4% vs. 37.7%). HPV-16/18 showed similar proportions (21.2% vs. 22.1%). Multiple HR-HPV infections were also more frequent in the persistent group (26.0% vs. 19.1%, P<0.001), whereas the proportion of mixed infections (concurrent HR-HPV and low-risk/intermediate-risk HPV) did not differ significantly between groups (18.5% vs. 17.1%, P = 0.346). Regarding vaginal dysbiosis, bacterial vaginosis distribution differed significantly (P = 0.018), with lower bacterial vaginosis prevalence in the persistent group (16.1% vs. 19.3%) and a lower proportion of intermediate bacterial vaginosis (15.1% vs. 17.1%), while normal status was more common in persistent infections (68.8% vs. 63.7%). aerobic vaginitis showed no significant difference between groups (23.5% vs. 24.8%, P = 0.440). vulvovaginal candidiasis was less common in persistent infections (9.6% vs. 13.5%, P = 0.002).

The distribution of HR-HPV subtype categories differed significantly between the persistent and transient groups (**Figure 2**). HPV-52/58 accounted for the largest proportion in both groups but was substantially more prevalent in the persistent group (51.4% vs. 40.2%, +11.2 percentage points), whereas other HR-HPV types (31, 33, 35, 39, 45, 51, 56, 59, 68) were less common in the persistent group (27.4% vs. 37.7%, −10.3 percentage points). HPV-16/18 showed comparable proportions between groups (21.2% vs. 22.1%) (**Figure 2**).

**Figure 2:**
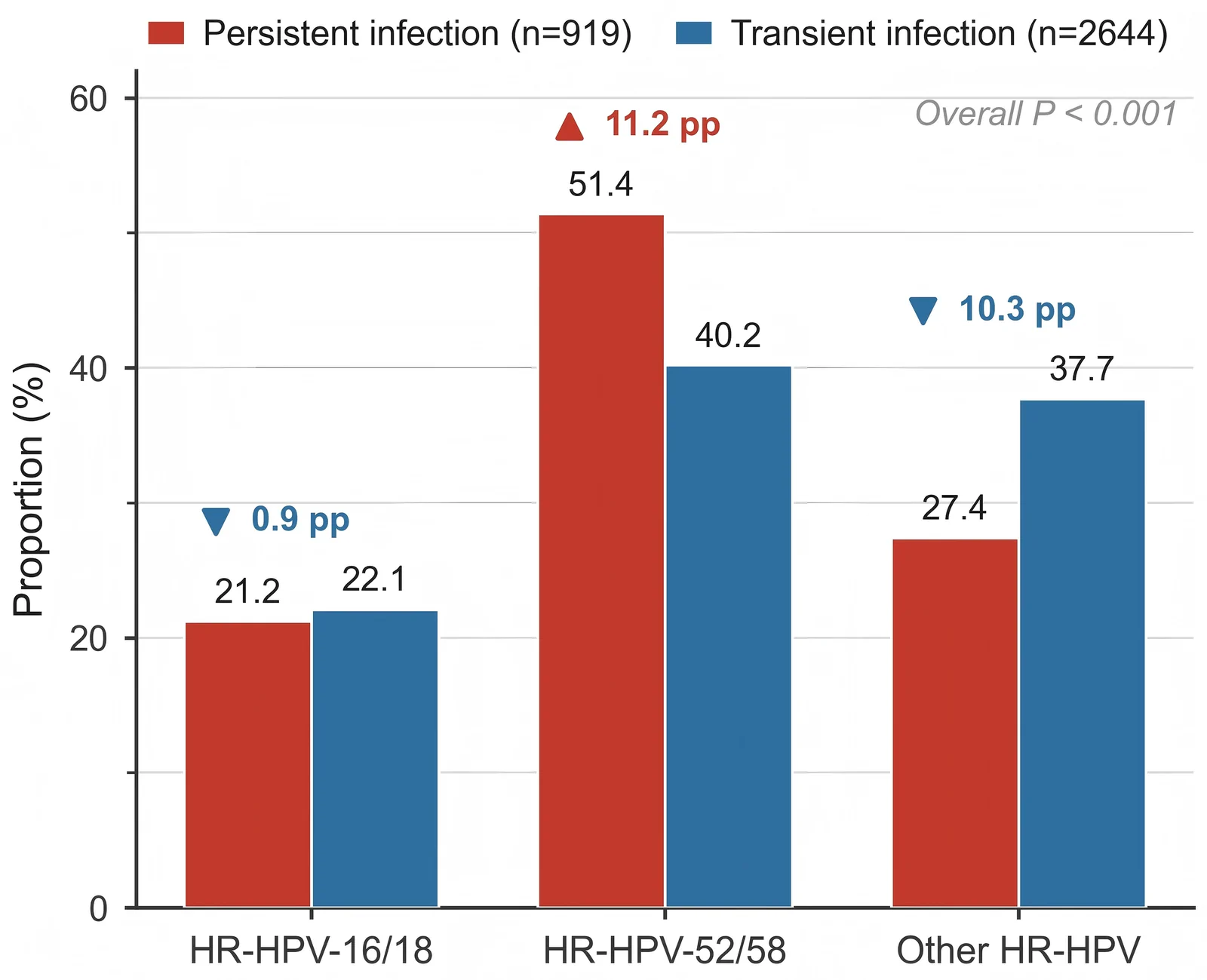
HR-HPV subtype distribution in persistent and transient infection groups. The bar chart shows the proportions of HPV-16/18, HPV-52/58, and other HR-HPV subtypes in the persistent infection group (n=919) and transient infection group (n=2,644). Data are presented as percentages of each subtype category within each group. Red upward arrows indicate subtypes with an increased proportion in the persistent group; blue downward arrows indicate subtypes with a decreased proportion. HR-HPV, high-risk human papillomavirus; pp, percentage points.

Univariate analysis (**Supplementary Table S4**) identified multiple factors associated with persistence, including HPV-16/18, HPV-52/58, multiple HR-HPV infections, bacterial vaginosis, vulvovaginal candidiasis, pH ≥4.6, H_2_O_2_ negativity, leukocyte esterase positivity, sialidase positivity, and LBG IIb–III (all P<0.05). Mixed infection, aerobic vaginitis, vaginal cleanliness, and overall microecological status showed no significant associations (**Supplementary Table S4**).

Multivariate logistic regression (**Supplementary Table S1**; **Figure 3**), adjusting for age, HR-HPV subtype category, number of HR-HPV infections, mixed infection, aerobic vaginitis, bacterial vaginosis, and vulvovaginal candidiasis, confirmed the independent role of age (per 5-year increase: OR = 1.13, 95% CI: 1.09–1.17, P<0.001), HPV-52/58 (OR = 1.65, 95% CI: 1.38–1.98, P<0.001), and multiple HR-HPV infections (OR = 1.50, 95% CI: 1.24–1.81, P<0.001) as independent risk factors for persistence. Of note, bacterial vaginosis (OR = 0.69, 95% CI: 0.55–0.88, P = 0.002) and intermediate bacterial vaginosis (OR = 0.66, 95% CI: 0.52–0.84, P<0.001) remained negatively associated with persistence, as did vulvovaginal candidiasis (OR = 0.75, 95% CI: 0.58–0.96, P = 0.024). HPV-16/18 showed a non-significant trend toward increased risk (OR = 1.21, 95% CI: 0.97–1.51, P = 0.088). Mixed infection (OR = 1.04, 95% CI: 0.85–1.28, P = 0.678) and aerobic vaginitis (OR = 1.11, 95% CI: 0.90–1.37, P = 0.348) were not independently associated with persistence. The inverse associations of bacterial vaginosis, intermediate bacterial vaginosis, and vulvovaginal candidiasis should not be interpreted as protective causal effects; bias analyses are presented in **Supplementary Table S8**.

**Figure 3:**
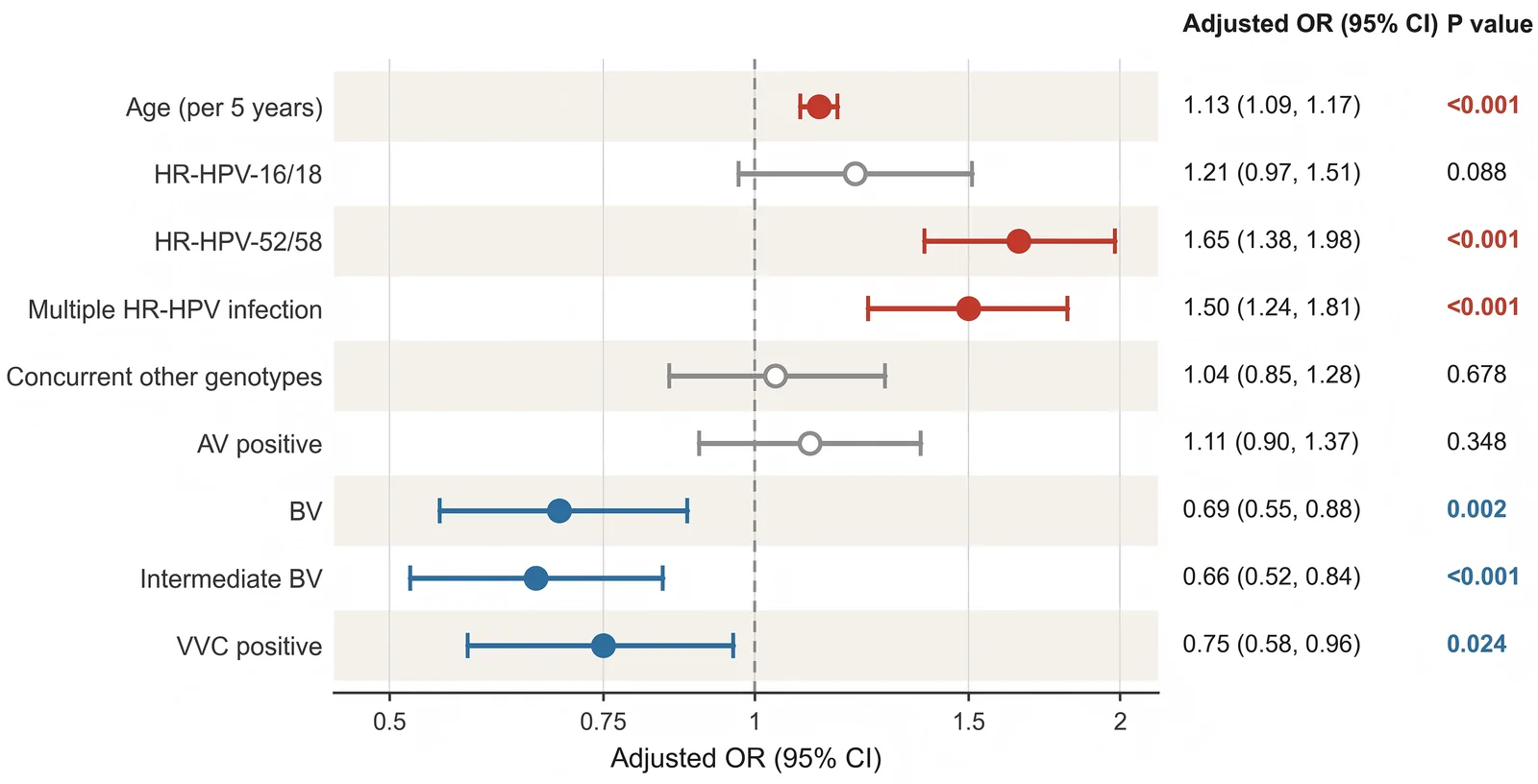
Forest plot of multivariate logistic regression results for factors associated with persistent HR-HPV infection. Adjusted odds ratios (ORs) with 95% confidence intervals (CIs) are plotted on a logarithmic scale. The model was adjusted for age (per 5-year increase), HPV subtype category (HPV-16/18 and HPV-52/58 vs. other HR-HPV), multiple HR-HPV infection (vs. single), mixed infection (vs. pure HR-HPV), aerobic vaginitis (vs. no aerobic vaginitis), bacterial vaginosis (intermediate bacterial vaginosis and bacterial vaginosis vs. normal), and vulvovaginal candidiasis (vs. no vulvovaginal candidiasis). Statistically significant factors (P<0.05) are highlighted in red. HR-HPV, high-risk human papillomavirus; OR, odds ratio; CI, confidence interval; AV, aerobic vaginitis; BV, bacterial vaginosis; VVC, vulvovaginal candidiasis.

Among the 919 persistently infected patients, total observed person-years was 1,951.3. aerobic vaginitis had the highest incidence density at 35.77 per 100 person-years (95% CI: 33.17–38.53), followed by bacterial vaginosis at 18.09 (95% CI: 16.25–20.08) and vulvovaginal candidiasis at 17.94 (95% CI: 16.11–19.92) (**Supplementary Table S2**; **Figure 4a**).

**Figure 4:**
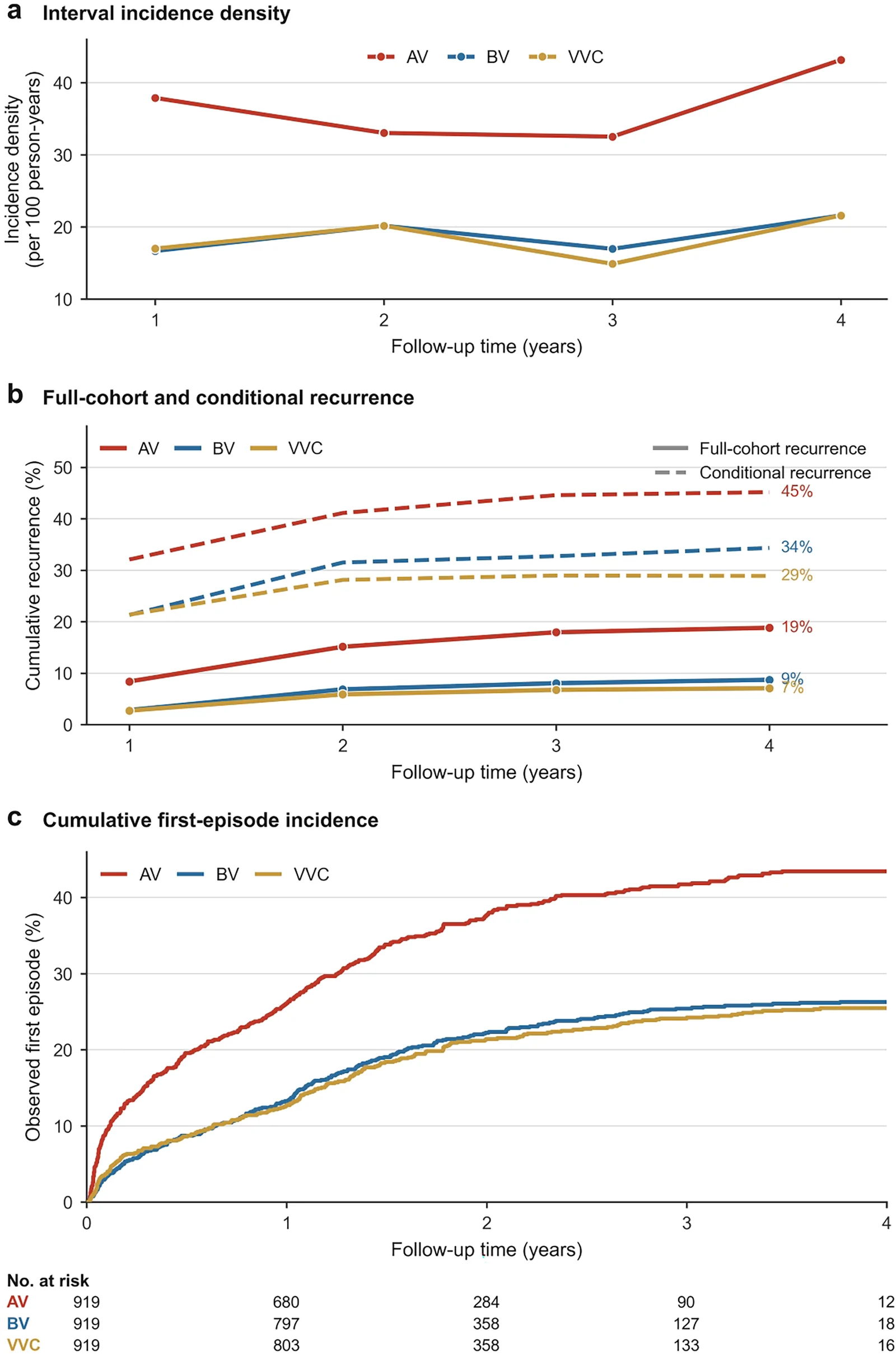
Temporal changes in aerobic vaginitis, bacterial vaginosis, and vulvovaginal candidiasis recurrence burden during follow-up in persistently infected patients. **(a)** Incidence density (episodes per 100 person-years) with 95% confidence intervals for aerobic vaginitis, bacterial vaginosis, and vulvovaginal candidiasis in the persistently infected cohort (n=919). aerobic vaginitis had the highest incidence density (35.77 per 100 PY). **(b)** Conditional recurrence rate (recurrence cases/patients with ≥1 episode) for each infection type. aerobic vaginitis exhibited the highest conditional recurrence rate (45.4%), followed by bacterial vaginosis (34.8%) and vulvovaginal candidiasis (28.9%). **(c)** Cumulative recurrence rates over 4-year follow-up in the full cohort. Shaded areas represent 95% confidence intervals. AV, aerobic vaginitis; BV, bacterial vaginosis; VVC, vulvovaginal candidiasis; PY, person-years.

At least one episode of aerobic vaginitis occurred in 383 patients (41.7%), with 174 (18.9%) experiencing recurrence. The conditional recurrence rate (recurrence among those with ≥1 episode) was 45.4% for aerobic vaginitis, substantially higher than bacterial vaginosis (34.8%) and vulvovaginal candidiasis (28.9%) (**Supplementary Table S5**; **Figure 4b**).

Sensitivity analyses using >60-day and >90-day new-episode thresholds showed consistent ranking, with aerobic vaginitis remaining the highest (**Supplementary Table S9**). At 4-year follow-up, full cohort recurrence rates reached 18.8% for aerobic vaginitis, 8.7% for bacterial vaginosis, and 7.1% for vulvovaginal candidiasis (**Figure 4c**). The proportion of patients experiencing their first episode by 365 days was 26.1% for aerobic vaginitis, 13.3% for bacterial vaginosis, and 12.7% for vulvovaginal candidiasis; by 730 days, these proportions increased to 37.6%, 22.2%, and 21.4%, respectively.

Negative binomial regression (**Supplementary Table S3**) revealed that mixed infection (HR-HPV with other types) was associated with higher bacterial vaginosis recurrence burden (IRR = 1.55, 95% CI: 1.15–2.10, P = 0.004). Increasing age was associated with lower vulvovaginal candidiasis recurrence burden (per 5 years: IRR = 0.78, 95% CI: 0.72–0.84, P<0.001). aerobic vaginitis recurrence showed a non-significant trend toward higher burden with mixed infection (IRR = 1.17, 95% CI: 0.90–1.51, P = 0.235).

TCT results during follow-up were evaluable in 3,416 of 3,563 patients (95.9%). The distribution of the highest TCT grade during follow-up is presented in **Supplementary Figure S1**. NILM was the most common outcome in both groups, occurring in 410 persistent cases (45.0%) compared with 1,459 transient cases (58.3%) (P<0.001). LSIL was more frequent in persistent infections (246, 27.0% vs. 450, 18.0%), while HSIL/ASC-H+ proportions were similar (5.9% vs. 5.9%) (**Supplementary Figure S1A**). Within the persistent group, stratification by HPV subtype (**Supplementary Figure S1B**) revealed that HPV-16/18 was associated with the highest proportion of HSIL/ASC-H+ lesions (approximately 7.2%), whereas HPV-52/58 and other HR-HPV subtypes showed lower proportions of high-grade cytological abnormalities.

Among 3,416 patients with evaluable TCT results (912 persistent, 2,504 transient), abnormal cytology (≥ASC-US) occurred in 1,547 patients, LSIL or higher lesions (LSIL+) in 897, and HSIL or ASC-H (HSIL/ASC-H+) in 201. Multivariable logistic regression (**Supplementary Table S5**), adjusted for age, HPV genotype, and vaginal microbiota parameters, showed that persistent infection was independently associated with ≥ASC-US (OR = 1.63, 95% CI: 1.40–1.91, P<0.001) and LSIL+ (OR = 1.48, 95% CI: 1.25–1.75, P<0.001), but not with HSIL/ASC-H+ (OR = 0.91, 95% CI: 0.66–1.27, P = 0.578).

To validate the robustness of our primary finding regarding HPV-52/58, we performed multiple restricted analyses (**Supplementary Table S6**; **Supplementary Figure S2**). The positive association between HPV-52/58 and persistence remained statistically significant and consistent across all analyses, including the original model (OR = 1.65, P<0.001), follow-up ≥180 days (OR = 1.63, P<0.001), follow-up ≥365 days (OR = 1.93, P<0.001), HR-HPV-only infections (OR = 1.71, P<0.001), single HR-HPV infections (OR = 1.71, P<0.001), and the persistent versus single-positive comparison (OR = 1.77, P<0.001). Importantly, the transient versus single-positive comparison showed no significant association (OR = 1.09, 95% CI: 0.97–1.21, P = 0.141), confirming the specificity of this finding to persistent infection.

## Discussion

### Summary of main results

In this retrospective cohort of 3,563 women with cervical LSIL, we identified HPV-52/58 (OR = 1.65), multiple HR-HPV infections (OR = 1.50), and advancing age (per 5 years: OR = 1.13) as independent risk factors for persistent HR-HPV infection. Notably, bacterial vaginosis and intermediate bacterial vaginosis showed negative associations with persistence, as did vulvovaginal candidiasis (OR = 0.75). Among persistently infected women, aerobic vaginitis had the highest recurrence burden (35.77 per 100 person-years), with a conditional recurrence rate of 45.4%, substantially exceeding bacterial vaginosis and vulvovaginal candidiasis. Persistent infection was independently associated with TCT abnormalities (OR = 1.63) and LSIL+ lesions (OR = 1.48).

### Results in the context of published literature

Our finding that HPV-52/58 is a robust independent risk factor for persistence aligns with previous studies demonstrating that these subtypes are the most prevalent high-risk HPV types among Chinese women and are characterized by lower natural clearance rates (Liu et al., 2025; Peng et al., 2025). Recent large-scale population studies in Western China (n=129,585) confirmed that non-vaccine-targeted HR-HPV subtypes HPV-52 and HPV-58 maintain sustained dominance across all age groups (Liu et al., 2025). The consistency of our sensitivity analyses (OR range 1.63–1.93 across six restricted datasets) reinforces the specificity of this association to persistent infection, as the transient vs. single-positive comparison showed no significant association.

The inverse associations of bacterial vaginosis (OR=0.69), intermediate bacterial vaginosis (OR=0.66), and vulvovaginal candidiasis (OR=0.75) with HPV persistence diverge from most prior studies, including a Finnish cohort (OR=2.15) (Kero et al., 2017) and a Kenyan prospective cohort (RR=1.38) (Chibvongodze et al., 2026), and earlier work on BV and HPV natural history (King et al., 2011). Direct comparisons are limited by differences in study populations, diagnostic methods, follow-up durations, and adjustment for confounders. Importantly, our bias analyses (**Supplementary Table S8**) indicated that these inverse associations are most likely attributable to detection-time bias and treatment-related behavior rather than to a protective biological effect: bacterial vaginosis-positive women were re-tested more frequently and were therefore more likely to be observed to clear; after excluding patients with short-interval re-testing as a proxy for immediate post-diagnosis management, the bacterial vaginosis and vulvovaginal candidiasis estimates moved toward the null and lost significance, whereas the HPV-52/58 and multiple-infection estimates were essentially unchanged. Reverse causality also cannot be excluded, because microecology and HPV were sampled at the same visit, and approximately half of the bacterial vaginosis/intermediate bacterial vaginosis episodes were already present at baseline. Residual confounding from unmeasured factors, including sexual behaviors, douching practices, antibiotic exposure, and HPV vaccination status, may also contribute. These inverse associations should therefore not be construed as evidence of a protective causal effect, and prospective studies with detailed treatment and vaccination records are needed.

An additional possibility is that patients diagnosed with bacterial vaginosis or vulvovaginal candidiasis received early antibiotic or antifungal therapy, which may have reduced pathogen burden and indirectly facilitated HPV clearance; because treatment data were not systematically captured, this explanation remains speculative and requires prospective validation.

Current guidelines recommend immediate colposcopy for HPV-16/18, whereas HPV-52/58 and other non-16/18 HR-HPV types are usually managed with observation and repeat testing (Zhao et al., 2022; Li et al., 2025a). This disparity could introduce confounding. HPV-16/18 infections, more often treated early, have a shorter window for persistence and fewer associated bacterial vaginosis/vulvovaginal candidiasis episodes. HPV-52/58 infections, observed longer, have a prolonged persistence period during which associated vaginal dysbiosis (particularly aerobic vaginitis) becomes more apparent. This would be consistent with our observation that aerobic vaginitis showed the highest recurrence burden in persistently infected women. This hypothesis is speculative without treatment-level and genotype-specific microecology data, and requires prospective validation.

The high recurrence burden of aerobic vaginitis in persistently infected women (35.77 per 100 person-years) is a novel finding that extends previous research. aerobic vaginitis is characterized by intense local inflammation and mucosal damage, and its promoting effect on HPV persistence appears stronger than that of bacterial vaginosis (Ji et al., 2023). Recent evidence demonstrates that persistent HPV infection is associated not only with bacterial vaginosis‑related pathogens but also with aerobic vaginitis‑related pathogens, with Gardnerella and Atopobium exhibiting synergistic co‑colonization (Liang et al., 2025; Tsementzi et al., 2025; Zeng et al., 2025). The high conditional recurrence rate of aerobic vaginitis (45.4%) suggests that once aerobic vaginitis is established in persistently infected patients, recurrence is highly likely without targeted intervention.

Regarding cervical cytology, persistent infection was significantly associated with TCT abnormalities (≥ASC-US: OR = 1.63) and LSIL+ lesions (OR = 1.48), but not HSIL/ASC-H+ (OR = 0.91). This pattern is consistent with the concept that at the LSIL stage, cytological abnormalities are more closely linked to HPV persistence, whereas high-grade lesions are more strongly associated with highly oncogenic subtypes such as HPV16 (Wang et al., 2021a; Zhao et al., 2025; Shen and Xu, 2023). Community-based longitudinal studies have confirmed that among CIN1 women, HPV58, 52, and 31 infections are more likely to resolve than HPV16, further supporting the differential natural history of HPV subtypes (Zhao et al., 2025; Li et al., 2025b).

### Strengths and weaknesses

Strengths of this study include the large sample size (n=3,563) in a well-characterized LSIL population, rigorous statistical approaches including multivariate adjustment and sensitivity analyses, and the novel quantification of recurrence burden for different vaginal infection types.

Several limitations should be considered. First, the retrospective design precludes establishing causal relationships between vaginal dysbiosis and HPV persistence. Prospective studies with sequential vaginal sampling are needed. Second, data were derived from a single tertiary referral center in Guangzhou, which may limit generalizability to other populations with different HPV genotype distributions and clinical practice patterns. Multi-center studies are needed. Third, treatment status (including antibiotic and antifungal use) was not systematically captured, potentially influencing observed associations. Fourth, median follow-up was approximately 2.0 years in the persistent group and 0.5 years in the transient group; this may not capture HSIL progression events that typically occur over longer time frames. Fifth, HPV vaccination status was not captured in this retrospective cohort and therefore represents an unmeasured confounder whose direction and magnitude cannot be determined from these data. During the study period (2021-2025) and in this region, overall vaccination coverage was likely low and dominated by bivalent/quadrivalent vaccines, but nine-valent coverage is increasing, particularly in younger cohorts; we therefore cannot assume that HPV-52/58 is unaffected. For the age-persistence association (OR = 1.13 per 5 years), vaccination status may confound the estimate: vaccination is more common in younger women, and vaccinated women may clear vaccine-covered types more rapidly and thus be classified as transient, which would inflate the apparent age effect; the age OR should therefore be interpreted as potentially overestimated. For the HPV-52/58 association, vaccinated individuals are less likely to acquire or maintain vaccine-covered types; if anything, this would attenuate the observed association toward the null, so the persistence of a robust association despite this potential attenuation strengthens, rather than weakens, the finding. The high HPV-52/58 prevalence in our cohort likely reflects the largely unvaccinated or under-vaccinated epidemiological context of the study period. Prospective studies with detailed vaccination histories are needed. Sixth, 16S rRNA sequencing was not performed because of the retrospective design. In addition, because the persistent definition requires an observed window of ≥365 days, the persistent group is by definition under observation through 365 days, which may introduce immortal time bias. We used t0 as the start of person-time for both groups and performed restricted analyses (follow-up ≥180 days and ≥365 days), but this bias cannot be fully eliminated, and it may inflate the observed recurrence burden of aerobic vaginitis in the persistent group.

### Implications for practice and future research

Based on our findings, we propose several practice recommendations for clinicians managing LSIL patients with HR-HPV infection. Women with HPV-52/58 or multiple concurrent HR-HPV infections warrant more intensive follow-up, given their 1.5- to 1.7-fold increased persistence risk. For this subgroup, we suggest shortening the surveillance interval to 6–12 months rather than the conventional 12-month repeat co-testing, to enable earlier detection of persistence and timely intervention.

We also recommend including standardized vaginal microecology testing, encompassing Nugent score, aerobic vaginitis score, and fungal evaluation, as part of the initial LSIL workup. Given the high recurrence burden of aerobic vaginitis in persistently infected women (35.77 per 100 person-years), this baseline assessment can identify patients at risk for recurrent dysbiosis and guide preventive strategies. The conditional recurrence rate of aerobic vaginitis (45.4%) further indicates that a single treatment episode is often insufficient. Clinicians should confirm microbiological cure after aerobic vaginitis treatment, schedule follow-up visits at 1–3 months post-treatment to detect early recurrence, and consider maintenance or prophylactic strategies for patients with documented aerobic vaginitis recurrence, particularly those with persistent HPV infection.

Although the inverse bacterial vaginosis association is likely non-causal, early identification and treatment of bacterial vaginosis in LSIL patients remains reasonable to reduce the overall burden of vaginal dysbiosis. However, it should not be presented as a strategy to promote HPV clearance, and treated patients should be reassessed for microbiological cure. Finally, advancing age was independently associated with persistence (per 5 years: OR = 1.13). Clinicians should communicate to older LSIL patients, particularly those aged 40 years or older, that age confers increased persistence risk, and should emphasize adherence to follow-up schedules. This age-specific counseling may improve patient engagement and reduce loss to follow-up in higher-risk subgroups. Additionally, although causal evidence is lacking, the high aerobic vaginitis recurrence burden and the known protective role of Lactobacillus-dominant communities suggest that probiotic or prebiotic interventions aimed at restoring vaginal eubiosis may be a reasonable adjunctive measure in persistently infected patients with recurrent dysbiosis. This approach should be considered investigational at present, but represents a promising direction for future clinical trials.

## Conclusions

This retrospective cohort study of 3,563 LSIL patients demonstrates that HPV-52/58, multiple HR-HPV infections, and advancing age are independent risk factors for persistent HR-HPV infection. The inverse associations of bacterial vaginosis, intermediate bacterial vaginosis, and vulvovaginal candidiasis with persistence are likely non-causal and should not be interpreted as protective effects, as they appear to reflect detection-time bias and treatment-related behavior. The high aerobic vaginitis recurrence burden in persistently infected women (35.77 per 100 person-years) underscores the need for integrated management strategies addressing both viral persistence and vaginal dysbiosis. These findings support personalized surveillance strategies based on HPV genotype, age, and vaginal microbiota status to improve clinical outcomes in LSIL patients through risk-stratified management.

## Data Availability

The data presented in the study are not publicly available due to hospital data privacy regulations. De-identified data may be obtained from the corresponding author upon reasonable request.

## Supplementary material

Supplementary Figure 1 TCT results distribution. **(A)** Distribution of the highest TCT grade during follow-up in persistent vs. transient infection groups. **(B)** Distribution of TCT grades stratified by HR-HPV subtype groups (HPV-16/18, HPV-52/58, and other HR-HPV) within the persistent infection group. TCT, ThinPrep cytology test; NILM, negative for intraepithelial lesion or malignancy; ASC-US, atypical squamous cells of undetermined significance; ASC-H, atypical squamous cells—cannot exclude HSIL; HSIL/ASC-H+, HSIL or ASC-H; LSIL, low-grade squamous intraepithelial lesion; HSIL, high-grade squamous intraepithelial lesion; HR-HPV, high-risk human papillomavirus.

Supplementary Figure 2 Sensitivity analysis forest plot for HPV-52/58 association with persistent infection. Adjusted odds ratios (ORs) with 95% confidence intervals (CIs) for HPV-52/58 (vs. other HR-HPV) across six restricted datasets: original model (OR = 1.65, P<0.001); follow-up ≥180 days (OR = 1.63, P<0.001); follow-up ≥365 days (OR = 1.93, P<0.001); HR-HPV-only infections (OR = 1.71, P<0.001); single HR-HPV infections (OR = 1.71, P<0.001); persistent vs. single-positive (OR = 1.77, P<0.001); and transient vs. single-positive (OR = 1.09, P = 0.141). The positive association remained significant across all analyses except the transient vs. single-positive comparison, confirming the specificity of this finding to persistent infection. All models were adjusted for age, multiple infections, and other confounders as appropriate. OR, odds ratio; CI, confidence interval; HR-HPV, high-risk human papillomavirus.

Supplementary Figure 3 Study design and testing timeline. t0 denotes the index date (first HR-HPV-positive test). The 30-day minimum interval distinguishes separate clinical episodes, and the 365-day threshold defines persistent infection. Red circles, HR-HPV-positive tests; white circles, HR-HPV-negative/cleared; squares, vaginal microecology (AV/BV/VVC) and cytology assessments. HR-HPV, high-risk human papillomavirus; AV, aerobic vaginitis; BV, bacterial vaginosis; VVC, vulvovaginal candidiasis.
