# Supplementary material for: Risk factors for persistent high-risk HPV infection and recurrence burden of vaginal infections in women with cervical low-grade squamous intraepithelial lesions: a retrospective cohort study
Source: Front Cell Infect Microbiol. 2026 Oct 22;16:1983448. doi: 10.3389/fcimb.2026.1983448 (PMC13735574; doi:10.3389/fcimb.2026.1983448)
Supplement: Supplementary file 1 [file Table1.docx]

Table S1. Multivariate Logistic Regression for Factors Associated with Persistent HR-HPV Infection

| **Variable** | **Level** | **Adjusted OR (95% CI)** | **P-value** |
| --- | --- | --- | --- |
| Age (per 5-year increase) |  | 1.13 (1.09, 1.17) | <0.001 |
| HR-HPV-16/18 | vs. other HR-HPV | 1.21 (0.97, 1.51) | 0.088 |
| HR-HPV-52/58 | vs. other HR-HPV | 1.65 (1.38, 1.98) | <0.001 |
| Multiple HR-HPV | vs. single | 1.50 (1.24, 1.81) | <0.001 |
| Mixed infection | vs. pure HR-HPV | 1.04 (0.85, 1.28) | 0.678 |
| aerobic vaginitis | vs. no aerobic vaginitis | 1.11 (0.90, 1.37) | 0.348 |
| bacterial vaginosis | vs. normal | 0.69 (0.55, 0.88) | 0.002 |
| Intermediate bacterial vaginosis | vs. normal | 0.66 (0.52, 0.84) | <0.001 |
| vulvovaginal candidiasis | vs. no vulvovaginal candidiasis | 0.75 (0.58, 0.96) | 0.024 |

Model included all variables listed; n=3,563

Adjusted odds ratios (ORs) and 95% confidence intervals (CIs) were derived from a multivariate logistic regression model including all variables listed in the table. The model was adjusted for age (per 5-year increase), HR-HPV subtypes (HR-HPV-16/18 and HR-HPV-52/58 vs. other HR-HPV), multiple HR-HPV infection (vs. single), mixed infection (vs. pure HR-HPV), aerobic vaginitis (vs. no aerobic vaginitis), bacterial vaginosis (intermediate bacterial vaginosis and bacterial vaginosis vs. normal), and vulvovaginal candidiasis (vs. no vulvovaginal candidiasis). Total sample size for the model was n=3,563. Statistically significant results (P<0.05) are indicated in bold. Abbreviations: OR, odds ratio; CI, confidence interval; HR-HPV, high-risk human papillomavirus; aerobic vaginitis, aerobic vaginitis; bacterial vaginosis, bacterial vaginosis; vulvovaginal candidiasis, vulvovaginal candidiasis.

Table S2. Recurrence Burden of aerobic vaginitis, bacterial vaginosis, and vulvovaginal candidiasis in Persistent Infection Patients

| **Type** | **Total n** | **Patients with ≥1 Episode** | **Total Episodes** | **Recurrence Cases** | **Full Cohort Recurrence Rate (%)** | **Conditional**  **Recurrence Rate (%)** | **Person-**  **Years** | **Incidence Density**  **(per 100 PY)** | **95% CI** |
| --- | --- | --- | --- | --- | --- | --- | --- | --- | --- |
| aerobic vaginitis | 919 | 383 | 698 | 174 | 18.9 | 45.4 | 1,951.30 | 35.77 | 33.17–38.53 |
| bacterial vaginosis | 919 | 233 | 353 | 81 | 8.8 | 34.8 | 1,951.30 | 18.09 | 16.25–20.08 |
| vulvovaginal candidiasis | 919 | 225 | 350 | 65 | 7.1 | 28.9 | 1,951.30 | 17.94 | 16.11–19.92 |

PY: person-years; Conditional recurrence rate = recurrence cases / patients with ≥1 episode

Total n = 919 persistently infected patients. Total observed person-years = 1,951.30. Conditional recurrence rate was calculated as recurrence cases divided by patients with ≥1 episode. Incidence density was calculated as total episodes divided by person-years, expressed per 100 person-years. A new episode of aerobic vaginitis, bacterial vaginosis, or vulvovaginal candidiasis was defined as a positive test occurring >30 days after the previous episode of the same type. Abbreviations: aerobic vaginitis, aerobic vaginitis; bacterial vaginosis, bacterial vaginosis; vulvovaginal candidiasis, vulvovaginal candidiasis; PY, person-years; CI, confidence interval.

Table S3. Negative Binomial Regression for Recurrence Burden

| **Types** | **Variable** | **Sample Size** | **IRR (95% CI)** | **P-value** |
| --- | --- | --- | --- | --- |
| aerobic vaginitis | Mixed with other types | 919 | 1.17 (0.90, 1.51) | 0.235 |
| bacterial vaginosis | Mixed with other types | 919 | 1.55 (1.15, 2.10) | 0.004 |
| vulvovaginal candidiasis | Age (per 5-year increase) | 919 | 0.78 (0.72, 0.84) | <0.001 |

Negative binomial regression models were fitted separately for aerobic vaginitis, bacterial vaginosis, and vulvovaginal candidiasis recurrence burden in persistently infected patients (n=919). Incidence rate ratios (IRRs) with 95% confidence intervals (CIs) are presented. Model for aerobic vaginitis and bacterial vaginosis: adjusted for mixed infection (vs. no) and age (per 5-year increase); model for vulvovaginal candidiasis: adjusted for mixed infection (vs. no) and age (per 5-year increase). Statistically significant results (P<0.05) are indicated in bold. Abbreviations: IRR, incidence rate ratio; CI, confidence interval; aerobic vaginitis, aerobic vaginitis; bacterial vaginosis, bacterial vaginosis; vulvovaginal candidiasis, vulvovaginal candidiasis.

Table S4. Univariate Logistic Regression Analysis of Factors Associated with Persistent HR-HPV Infection

| **Variable** | **Level** | **Persistent (n=919)** | **Transient (n=2,644)** | **Univariate OR (95% CI)** | **P-value** |
| --- | --- | --- | --- | --- | --- |
| **Age (per 5-year increase)** | Per 5-year increase | — | — | 1.11 (1.07, 1.15) | <0.001 |
| **HPV subtype category** |  |  |  |  |  |
|  | Other HR-HPV (ref) | 252 (27.4%) | 996 (37.7%) | 1.00 (Ref) |  |
|  | HPV-16/18 | 195 (21.2%) | 585 (22.1%) | 1.32 (1.06, 1.63) | 0.011 |
|  | HPV-52/58 | 472 (51.4%) | 1,063 (40.2%) | 1.76 (1.47, 2.09) | <0.001 |
| **Number of HR-HPV types** |  |  |  |  |  |
|  | Single (ref) | 680 (74.0%) | 2,140 (80.9%) | 1.00 (Ref) |  |
|  | Multiple | 239 (26.0%) | 504 (19.1%) | 1.49 (1.25, 1.78) | <0.001 |
| **Mixed infection** |  |  |  |  |  |
|  | Pure HR-HPV (ref) | 749 (81.5%) | 2,193 (82.9%) | 1.00 (Ref) |  |
|  | HR-HPV + low-risk/other | 170 (18.5%) | 451 (17.1%) | 1.10 (0.91, 1.34) | 0.321 |
| **Aerobic vaginitis (AV)** |  |  |  |  |  |
|  | No (ref) | 703 (76.5%) | 1,987 (75.2%) | 1.00 (Ref) |  |
|  | Yes | 216 (23.5%) | 657 (24.8%) | 0.93 (0.78, 1.11) | 0.414 |
| **Bacterial vaginosis (BV)** |  |  |  |  |  |
|  | Normal (ref) | 632 (68.8%) | 1,683 (63.7%) | 1.00 (Ref) |  |
|  | Intermediate | 139 (15.1%) | 452 (17.1%) | 0.82 (0.66, 1.01) | 0.064 |
|  | BV | 148 (16.1%) | 509 (19.3%) | 0.77 (0.63, 0.95) | 0.014 |
| **Vulvovaginal candidiasis (VVC)** |  |  |  |  |  |
|  | No (ref) | 831 (90.4%) | 2,286 (86.5%) | 1.00 (Ref) |  |
|  | Yes | 88 (9.6%) | 358 (13.5%) | 0.68 (0.53, 0.87) | 0.002 |
| **Vaginal pH** |  |  |  |  |  |
|  | 3.8–4.5 (ref) | 378 (41.1%) | 970 (36.7%) | 1.00 (Ref) |  |
|  | ≥4.6 | 539 (58.7%) | 1,673 (63.3%) | 0.83 (0.71, 0.96) | 0.015 |
| **Hydrogen peroxide (H₂O₂)** |  |  |  |  |  |
|  | Positive (ref) | 746 (81.2%) | 2,234 (84.5%) | 1.00 (Ref) |  |
|  | Negative | 172 (18.7%) | 410 (15.5%) | 1.26 (1.03, 1.53) | 0.023 |
|  | Weak | 1 (0.1%) | 0 (0.0%) | NE | 0.251 |
| **Leukocyte esterase** |  |  |  |  |  |
|  | Negative (ref) | 27 (2.9%) | 142 (5.4%) | 1.00 (Ref) |  |
|  | Positive | 651 (70.8%) | 1,984 (75.0%) | 1.73 (1.13, 2.63) | 0.011 |
|  | Weak | 241 (26.2%) | 518 (19.6%) | 2.45 (1.58, 3.80) | <0.001 |
| **Sialidase** |  |  |  |  |  |
|  | Negative (ref) | 795 (86.5%) | 2,155 (81.5%) | 1.00 (Ref) |  |
|  | Positive/Weak | 124 (13.5%) | 489 (18.5%) | 0.69 (0.56, 0.85) | <0.001 |
| **Vaginal cleanliness** |  |  |  |  |  |
|  | Grade I–II (ref) | 522 (56.8%) | 1,417 (53.6%) | 1.00 (Ref) |  |
|  | Grade III–IV | 397 (43.2%) | 1,227 (46.4%) | 0.88 (0.75, 1.02) | 0.092 |
| **LBG grade** |  |  |  |  |  |
|  | I–IIa (ref) | 509 (55.4%) | 1,322 (50.0%) | 1.00 (Ref) |  |
|  | IIb–III | 410 (44.6%) | 1,322 (50.0%) | 0.81 (0.69, 0.94) | 0.005 |
| **Overall microecological status** |  |  |  |  |  |
|  | Balanced (ref) | 184 (20.0%) | 480 (18.2%) | 1.00 (Ref) |  |
|  | Dysbiosis | 735 (80.0%) | 2,164 (81.8%) | 0.89 (0.73, 1.07) | 0.211 |

Data are presented as n (%) unless otherwise indicated. OR, odds ratio; CI, confidence interval; HR-HPV, high-risk human papillomavirus; AV, aerobic vaginitis; BV, bacterial vaginosis; VVC, vulvovaginal candidiasis; LBG, Lactobacillus grade; NE, not estimable. Mixed infection was defined as concurrent HR-HPV and low-risk or intermediate-risk HPV infection. BV classification: Normal (Nugent score 0–3), Intermediate (Nugent score 4–6), BV (Nugent score 7–10). LBG grade: I (Lactobacillus dominant), IIa (Lactobacillus predominant with mixed flora), IIb (reduced Lactobacillus), III (Lactobacillus severely reduced or absent). P-values were calculated using chi-square test or Fisher's exact test as appropriate. Statistically significant results (*P*<0.05) are indicated in bold.

Table S5. Logistic Regression for TCT Outcomes

| **Clinical Outcome** | **Sample Size** | **Events** | **Adjusted OR (95% CI)** | **P-value** |
| --- | --- | --- | --- | --- |
| TCT abnormal (≥ASC-US) | 3,416 | 1,547 | 1.63 (1.40, 1.91) | <0.001 |
| LSIL+ | 3,416 | 897 | 1.48 (1.25, 1.75) | <0.001 |
| HSIL/ASC-H+ | 3,416 | 201 | 0.91 (0.66, 1.27) | 0.578 |

Models were adjusted for age, HPV genotype, and vaginal microbiota parameters. Abbreviations: TCT, ThinPrep cytology test; ASC-US, atypical squamous cells of undetermined significance; LSIL, low-grade squamous intraepithelial lesion; HSIL, high-grade squamous intraepithelial lesion; OR, odds ratio; CI, confidence interval.

Table S6. Sensitivity Analysis for HR-HPV-52/58 Association with Persistent Infection

| **Analysis Set** | **Sample Size** | **Adjusted OR (95% CI)** | **P-value** |
| --- | --- | --- | --- |
| Original model | 3,563 | 1.65 (1.38, 1.98) | <0.001 |
| Follow-up ≥180 days | 2,309 | 1.63 (1.34, 1.99) | <0.001 |
| Follow-up ≥365 days | 1,607 | 1.93 (1.52, 2.45) | <0.001 |
| HR-HPV only | 2,942 | 1.71 (1.41, 2.08) | <0.001 |
| Single HR-HPV | 2,820 | 1.71 (1.41, 2.08) | <0.001 |
| Persistent vs. single positive | 5,372 | 1.77 (1.50, 2.10) | <0.001 |
| Transient vs. single positive | 7,097 | 1.09 (0.97, 1.21) | 0.141 |

All models were adjusted for age and other confounders as appropriate. Abbreviations: HR-HPV, high-risk human papillomavirus; OR, odds ratio; CI, confidence interval.

Table S7. Testing frequency, testing interval, and follow-up by group and by bacterial vaginosis status.

| Stratum | n | Median number of tests (IQR) | Median testing interval, days (IQR) | P value |
| --- | --- | --- | --- | --- |
| Persistent HR-HPV infection | 919 | 5 (4–8) | 184 (122–282) | <0.001^*^ |
| Transient HR-HPV infection | 2,644 | 4 (3–5) | 96 (45–167) |  |
| Bacterial vaginosis-positive | 657 | 4 | 93 | <0.001^†^ |
| Bacterial vaginosis-negative | 2,906 | 4 | 125 |  |

^*^Persistent (n=919) vs. transient (n=2,644) infection.
^†^Bacterial vaginosis-positive (n=657) vs. bacterial vaginosis-negative (n=2,906).
Data are presented as median (IQR). Comparisons were performed using the Mann–Whitney U test. HR-HPV, high-risk human papillomavirus; IQR, interquartile range; BV, bacterial vaginosis.

Table S8. Multivariable model after excluding patients with short-interval re-testing (proxy for immediate post-diagnosis management).

| Variable | Main model OR (95% CI) | Excluding ≤30 days OR (95% CI) | Excluding ≤45 days OR (95% CI) | Excluding ≤60 days OR (95% CI) |
| --- | --- | --- | --- | --- |
| HR-HPV-52/58 | 1.66 (1.38–1.98) | 1.67 (1.39–2.00) | 1.70 (1.41–2.05) | 1.75 (1.44–2.12) |
| Multiple HR-HPV infection | 1.50 (1.24–1.81) | 1.53 (1.26–1.85) | 1.57 (1.29–1.92) | 1.61 (1.31–1.98) |
| Bacterial vaginosis | 0.69 (0.55–0.88) | 0.79 (0.62–1.00) | 0.77 (0.60–0.99) | 0.78 (0.60–1.01) |
| Intermediate bacterial vaginosis | 0.66 (0.52–0.84) | 0.72 (0.56–0.92) | 0.70 (0.55–0.90) | 0.68 (0.53–0.89) |
| Vulvovaginal candidiasis | 0.75 (0.58–0.96) | 0.84 (0.65–1.09) | 0.86 (0.66–1.12) | 0.88 (0.67–1.16) |

The proxy indicator was a short mean testing interval, suggesting immediate post-diagnosis management or reassessment; it is not a record of actual treatment and should be regarded as an approximate sensitivity analysis only. HR-HPV, high-risk human papillomavirus; OR, odds ratio; CI, confidence interval.

Table S9. Recurrence metrics for aerobic vaginitis, bacterial vaginosis, and vulvovaginal candidiasis under different new-episode thresholds.

| New-episode threshold | Aerobic vaginitis conditional recurrence rate (%) | Bacterial vaginosis conditional recurrence rate (%) | Vulvovaginal candidiasis conditional recurrence rate (%) | Aerobic vaginitis incidence density (per 100 person-years) |
| --- | --- | --- | --- | --- |
| >30 days | 45.4 | 34.8 | 28.9 | 35.8 |
| >60 days | 42.3 | 33 | 25.8 | 32.9 |
| >90 days | 40.2 | 29.6 | 23.1 | 31.5 |

New episodes were defined using >30-day, >60-day, and >90-day thresholds between consecutive positive tests of the same type. Conditional recurrence rate = recurrence cases / patients with ≥1 episode. AV, aerobic vaginitis; BV, bacterial vaginosis; VVC, vulvovaginal candidiasis; PY, person-years.
